# Effects of dietary fucoxanthin on cholesterol metabolism in diabetic/obese KK-*A*^*y*^ mice

**DOI:** 10.1186/1476-511X-11-112

**Published:** 2012-09-10

**Authors:** Fumiaki Beppu, Masashi Hosokawa, Yoshimi Niwano, Kazuo Miyashita

**Affiliations:** 1 Faculty of Fisheries Sciences, Hokkaido University, 3-1-1 Minato Hakodate, Hokkaido, 041-8611, Japan; 2 Laboratory for Redox Regulation, Tohoku University Graduate School of Dentistry, 4-1 Seiryo-cho, Aoba-ku, Sendai, 980-8575, Japan

**Keywords:** Fucoxanthin, Cholesterol metabolism, Liver, Sterol regulatory element binding protein (SREBP), Low-density lipoprotein (LDL) receptor (LDLR), Scavenger receptor class B type 1 (SR-B1), Proprotein convertase subtilisin/kexin type 9 (PCSK9)

## Abstract

**Background:**

Fucoxanthin is a xanthophyll present in brown seaweeds and has several beneficial effects, including anti-obesity and anti-diabetic effects. However, we and another group previously observed that fucoxanthin increases serum cholesterol levels in rodents. Cholesterol is an important component of cell membranes and biosynthesis of bile acids. Serum cholesterol levels are also closely associated with atherosclerosis. Therefore, we sought to identify the mechanism underlying the increase in serum cholesterol levels by fucoxanthin.

**Methods:**

Diabetic/obese KK-*A*^*y*^ mice were fed a diet containing 0.2% fucoxanthin for 4 weeks. The mice were sacrificed, and total blood samples were collected for the measurement of serum total cholesterol, HDL-cholesterol and non-HDL-cholesterol levels. Cholesterol content in tissues was also analyzed. Real-time PCR and Western blotting were performed to determine hepatic mRNA and protein expression of genes involved in cholesterol metabolism, respectively.

**Results:**

Dietary fucoxanthin significantly increased serum HDL and non-HDL cholesterol levels, and reduced hepatic cholesterol content. In liver, the expression of SREBP1, SREBP2 and their target genes involved in cholesterol biosynthesis significantly increased and tended to increase in the fucoxanthin-fed mice, respectively. In contrast, hepatic levels of LDLR and SR-B1 proteins which is important factors for LDL-cholesterol and HDL-cholesterol uptake in the liver from serum, decreased to 60% and 80% in the fucoxanthin-fed mice, respectively, compared with the control mice. Further, we found that dietary fucoxanthin significantly increased the mRNA expression of proprotein convertase subtilisin/kexin type 9 (PCSK9), which enhances intracellular degradation of LDLR in lysosomes.

**Conclusions:**

Fucoxanthin increased HDL-cholesterol and non-HDL-cholesterol levels in KK-*A*^*y*^ mice by inducing SREBP expression and reduced cholesterol uptake in the liver via down-regulation of LDLR and SR-B1, resulted in increased serum cholesterol in the mice.

## Introduction

Fucoxanthin is an orange-colored carotenoid present in edible brown seaweeds, such as *Undaria pinnatifida* and *Hijikia fusiformis*. Fucoxanthin has reported to exhibit beneficial health effects, such as anti-cancer [1], anti-inflammatory [2], and radical scavenging [3] activities. We previously reported that fucoxanthin suppresses body weight gain and ameliorates hyperglycemia in diabetic/obese KK-*A*^*y*^ mice [4].

On the other hand, Kadekaru et al. reported that oral fucoxanthin administration for 28 days significantly increased serum total-cholesterol levels in male and female rats [5]. We also observed an increase in serum total cholesterol levels in ICR mice administered a high dose of fucoxanthin (500 mg/kg body weight) for 30 days [6]. In contrast, Woo et al. reported that fucoxanthin (0.2% in diet) decreased plasma cholesterol levels in C57BL/6N mice fed a high-fat diet [7]. Thus, the effects of fucoxanthin on serum cholesterol levels are not clear. In addition, the effects of fucoxanthin on regulation of cholesterol transport and metabolism have not been investigated in detail.

Cholesterol is an essential component of plasma membranes and lipoproteins and is a precursor of internal steroids, such as corticosteroids, sex hormones, bile acids, and vitamin D. Cholesterol levels in the blood and tissues are regulated by a balance between biosynthesis and catabolism, which primarily involve the activities of 3-hydroxy-3-methyl-glutaryl-CoA (HMG-CoA) reductase and cholesterol 7 α-hydroxyrase (CYP7A1), respectively. The expression of genes involved in cholesterol synthesis is strictly regulated by sterol regulatory element binding protein (SREBP) 1 and SREBP2. In addition, low-density lipoprotein (LDL) receptor (LDLR), scavenger receptor class B type 1 (SR-B1), and ATP-binding cassette transporter A1 (ABCA1) play crucial roles in the uptake and efflux of cholesterol in the liver and peripheral tissues to control the cholesterol levels in the body.

In the present study, we investigated the effects of fucoxanthin on the molecular mechanism underlying cholesterol metabolism in diabetic/obese KK-*A*^*y*^ mice. Dietary fucoxanthin (0.2% in the diet) increased HDL-cholesterol and non-HDL-cholesterol levels as well as total cholesterol levels in the serum of KK-*A*^*y*^ mice. Hepatic SREBP2 expression was also increased by fucoxanthin, although hepatic cholesterol content decreased. Fucoxanthin resulted in reduced LDLR expression through up-regulation of hepatic expression of PCSK9 mRNA. In addition, the SR-B1 expression level in the liver was also decreased by fucoxanthin. Our results reveal that fucoxanthin affects on cholesterol metabolism and transport system, which alters the cholesterol balance among the serum, liver, and peripheral tissues.

## Materials and methods

### Materials

Dried brown seaweed, *Undaria pinnatifida*, was purchased from a market in Hakodate, Hokkaido in Japan. Anti-SREBP2, anti-LDLR and anti-ABCA1 antibodies were obtained from Abcam (Cambridge, MA, USA). Anti-SREBP1 and anti-SR-B1 were purchased from Santa Cruz Biotechnology (Santa Cruz, California, USA).

### Fucoxanthin preparation

Crude lipid containing fucoxanthin was extracted from dried *Undaria pinnatifida* powder with acetone. Fucoxanthin was purified by silicagel column chromatography with *n*-hexane/acetone (7:3, v/v) from the crude lipid as our previous report [8]. Purity of fucoxanthin (all-*trans*- and *cis*- fucoxanthin) was >95% by HPLC analysis.

### Animals and diet

Diabetic/obese KK-*A*^*y*^ mice (4-week old male) were purchased from CLEA Japan, Inc. (Tokyo, Japan). The mice had free access to drinking water and diet at 23 ± 1°C and 50% humidity with a 12 h light/12 h dark cycle. The diet was prepared according to the recommendations of American Institute of Nutrition (AIN-93G). After acclimation for a week by feeding AIN-93G, KK-*A*^*y*^ mice were assigned to two groups and fed the control diet (AIN-93G) and fucoxanthin (0.2%) diet for 4 weeks (Table 1). Blood samples were taken from caudal vena cava of the mice fasted for 12 hour under ether anesthesia. Then, liver, epididymal WAT, and skeletal muscle were removed, weighed and immediately frozen in liquid nitrogen for lipid analysis and Western blotting, or soaked in RNA later^TM^ (Sigma Chemical Co., St. Louis, MO) for quantitative real time PCR analysis. All procedures for the use and care of animals for this research were approved by the Ethical Committee of Experimental Animal Care at Hokkaido University.

**Table 1:** Composition of experiment diets based on AIN-93G

**Ingredient**	**Control (g/kg)**	**Fucoxanthin**
Corn starch	397.486	397.486
Casein	200	200
Sucrose	100	100
Dextrinized corn starch	132	132
Soybean oil	70	68
Cellulose	50	50
Mineral mix	35	35
Vitamin mix	10	10
L-Cystine	3	3
Coline bitartrate	2.5	2.5
*tert*-Burtylhydroquinone	0.014	0.014
Fucoxanthin	–	2.0

### Serum and tissue cholesterol analysis

Serum total-cholesterol and HDL-cholesterol levels were measured by each commercial assay kit (Wako pure chemicals, Osaka, Japan) according to the manufacturer’s instruction. Non-HDL-cholesterol was calculated by subtraction of HDL-cholesterol from total-cholesterol. Cholesterol content in tissue was measured by using a technique as described by Jeon SM et al. [9]. In brief, tissue lipids were extracted by Folch method [10]. An approximately 5 mg lipid was dissolved in 20 μl methanol/Triton X-100 (1:1, v/v) and methanol was removed by nitrogen gas. Distilled-deionized water was then added and mixed until the solution was clear. Cholesterol content was measured using commercial enzymatic assay kit (Wako Pure Chemical Industries, Ltd., Osaka, Japan) according to the manufacturer’s instruction.

### Quantitative real-time RT-PCR

Total RNA was isolated from the liver soaked in RNAlater with the RNeasy Mini Kit (Qiagen, Tokyo, Japan), according to the manufacturer’s protocol. Then, cDNA was synthesized from total RNA using High Capacity cDNA Reverse Transcription Kit (Applied Biosystems Japan Ltd., Tokyo, Japan). Quantitative real-time PCR analysis was carried out using the ABI Prism 7500 (Applied Biosystems Japan Ltd., Tokyo, Japan) with the FastStart Universal Probe Master (Rox) (Roche Diagnostics Japan Ltd., Tokyo, Japan). The mRNA expression levels were measured using the Taqman® Gene Expression Assays (Applied Biosystems Japan Ltd, Tokyo, Japan); Hmgcr: Mm01282499_m1, Hmgcs: Mm01304569_m1, Fdps: Mm00836315_g1, Lss: Mm00461312_m1, Cyp51:Mm00490968_m1, Cyp7a1: Mm00484152_m1, PCSK9: Mm01263610_m1 and β-Actin: Mm00607939_s1.

### Western blotting

Liver was suspended in 10 volumes of a buffer containing 50 mM Hepes, 150 mM NaCl, 1 mM EDTA, 2 mM EGTA, 50 mM sodium fluoride, 1% Triton X-100 and 1 mM phenylmethylsulfonyl fluoride supplemented protease inhibitor cocktail (Sigma Chemical Co., St. Louis, MO, USA). After centrifugation at 8,550 g for 60 min at 4°C, the supernatant was collected for assay. Protein concentration in the supernatant was determined by a colorimetric method using a DC protein assay kit (Bio-Rad Laboratories, Hercules, CA, USA). The lysates were mixed with the same volume of loading buffer (Sigma Chemical Co., St. Louis, MO, USA), and boiled for 5 min. Protein samples were loaded at 30 μg protein per lane, and separated by SDS-polyacrylamide (10%) gel electrophoresis. Thereafter, proteins were transferred onto a polyvinylidene difluoride membrane. The membrane was blocked with 0.5% non-fat dry milk in TBS buffer for 1 hour at room temperature. For detection of protein, the membranes were incubated with primary anti-bodies for 1 hour at room temperature. After a washing procedure, the membrane was incubated with horseradish peroxidase-conjugated secondary antibody (Santa Cruz Biotechnology, Inc., Santa Cruz, California, USA) for 1 hour at room temperature. The immunoreactive bands were visualized with ECL Plus Western Blotting Detection System (GE Healthcare Japan Corporation, Japan). Quantitative densitometric analyses were performed on digitized images of immunoblots using cooled CCD camera system (ATTO corporation, Tokyo, Japan). The β-Actin was used for loading standardization.

### Statistical analysis

Data are presented as the mean ± SEM. The statistical significance between control and fucoxanthin groups was analyzed by Student’s *t*-tests for each experiment.

## Results

### Cholesterol level in the serum, liver, white adipose tissue and muscle

Fucoxanthin (0.2% in AIN-93G diet) suppressed body weight gain of diabetic/obese KK-*A*^*y*^ mice as previous papers [4]. Food intake of fucoxanthin-fed mice tended to be lower, but not significantly, than that of control mice. Dietary fucoxanthin significantly increased serum total cholesterol levels in the KK-*A*^*y*^ mice (Table 2). HDL-cholesterol and non-HDL-cholesterol levels also increased in the fucoxanthin-fed mice. In contrast, hepatic cholesterol content was significantly lower in the fucoxanthin-fed mice than in the control mice (Table 3), although liver weight increased by fucoxanthin as our previous study [6]. These effects of fucoxanthin were associated with the amount of cholesterol per unit protein in the liver of the fucoxanthin-fed (26.36 ± 0.91) and control mice (50.59 ± 5.46), respectively. The mechanism by which fucoxanthin impacts on the liver is unclear.

**Table 2:** Total, HDL, and non-HDL cholesterol concentrations in the serum

	**Control (mg/dl)**	**Fucoxanthin**
Total cholesterol	119.0 ± 10.3	169.2 ± 7.3^**^
HDL cholesterol	85.6 ± 5.2	109.1 ± 6.9^*^
Non-HDL cholesterol	33.4 ± 9.1	60.1 ± 4.9^*^

KK-*A*^*y*^ mice were fed with the AIN-93G diet with or without 0.2% fucoxanthin for 4 weeks. Total and HDL-cholesterol was measured by an enzymatic method. Non-HDL-cholesterol was calculated by subtracting HDL-cholesterol from total-cholesterol. Each value represents the mean ± standard error (n = 6). Asterisk show significant differences from the control group (*P < 0.05<, **P < 0.01).

**Table 3:** Tissue weights and cholesterol contents in the liver, epididymal WAT, and muscle

		**Control**	**Fucoxanthin**
Liver	Weight (g)	1.62 ± 0.05	1.87 ± 0.05^**^
	Cholesterol (mg/liver)	11.10 ± 0.89	6.58 ± 0.19^**^
Epididymal WAT	Weight (g)	1.44 ± 0.12	0.99 ± 0.10^*^
	Cholesterol (mg/epididymal WAT)	14.76 ± 1.33	10.26 ± 0.96^**^
Muscle	Weight (g)	0.23 ± 0.01	0.23 ± 0.01
	Cholesterol (mg/muscle)	0.23 ± 0.02	0.23 ± 0.01

KK-*A*^*y*^ mice were fed with the AIN-93G diet with or without 0.2% fucoxanthin for 4 weeks. Cholesterol contents in tissues were measured by an enzymatic methods as shown in “Materials and Methods”. Each value represents the mean ± standard error (n = 6). Asterisks show significant differences from the control group (*P < 0.05<, **P < 0.01).

The total amount of cholesterol that had accumulated in epididymal WAT was also lower in the fucoxanthin-fed mice than in the control mice because epididymal WAT weight in the fucoxanthin-fed mice was lower than that in the control mice (Table 3). On the other hand, cholesterol level in skeletal muscle was not altered by fucoxanthin. These results show that fucoxanthin reduces the total cholesterol accumulation in the liver and WAT.

### Gene and protein expressions involved in cholesterol metabolism in the liver

**Figure 1:**
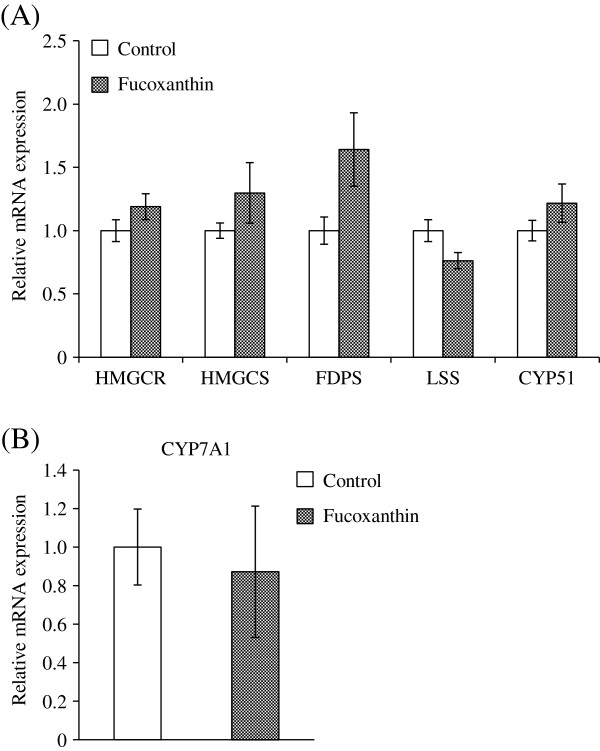
**Hepatic gene expression involved in cholesterol metabolism.** KK-*A*^*y*^ mice were fed with the AIN-93G diet with or without 0.2% fucoxanthin for 4 weeks. Total RNA was extracted from the liver and processed for quantitative real-time PCR analysis as described under “Materials and Methods”. The expression levels of each mRNA are normalized to β-actin levels. Data are presented relative to the control group. Each value represents the mean ± standard error (n = 6). HMGCR: HMG-CoA reductase, HMGCS: HMG-CoA synthase, FDPS: farnesyl diphosphate synthase, LSS: lanosterol synthase, CYP51: lanosterol 14α-demethylase, CYP7A1: cholesterol 7 α-hydroxyrase.

The expression levels of genes related to cholesterol biosynthesis and catabolism in the liver were analyzed by real-time quantitative PCR (Figure 1A). The mRNA expression level of HMG-CoA reductase (HMGCR), a rate-limiting enzyme in cholesterol synthesis, tended to increase in the fucoxanthin group. In addition, except that of lanosterol synthase (LSS), the mRNA expression level of key enzymes involved in cholesterol biosynthesis, including HMG-CoA synthase (HMGCS), farnesyl diphosphate synthase (FDPS), and lanosterol 14α-demethylase (CYP51), tended to be increased by fucoxanthin. In contrast, the mRNA expression level of CYP7A1, an enzyme associated with synthesis of bile acids from cholesterol, did not increase by fucoxanthin-diet (Figure 1B). Overall, the mRNA expression of enzymes involved in cholesterol synthesis tended to be higher in the fucoxanthin-fed mice, although not significantly.

**Figure 2:**
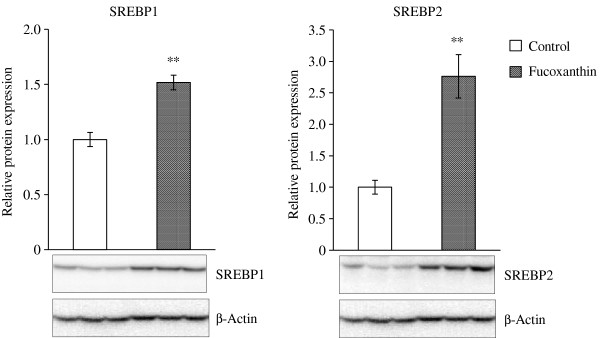
**SREBP1 and SREBP2 expressions in the liver.** KK-*A*^*y*^ mice were fed with the AIN-93G diet with or without 0.2% fucoxanthin for 4 weeks. Liver lysates were prepared for Western blotting, and primary antibody binding was detected by appropriate secondary antibodies for chemiluminescence as described under “Materials and Methods”. SREBP1 and SREBP2 levels are presented relative to control group after normalization for β-Actin levels. Each value represents the mean ± standard error (n = 6). Asterisks show significant differences from the control group (** P < 0.01).

We further examined the expression levels of SREBP1 and SREBP2, which are key transcriptional factors for the regulation of cholesterol metabolism. As shown in Figure 2, dietary fucoxanthin resulted in a 2.7-fold increase in SREBP2 expression compared with control mice. SREBP1 expression was also significantly increased in the fucoxanthin-fed mice. In particular, SREBP2 is known to be sensitively activated and to enhance gene transcription for cholesterol synthesis when cellular cholesterol levels decline [11,12]. Our results show that SREBP2 and SREBP1 expression was enhanced in the liver by fucoxanthin-diet, although hepatic cholesterol content in the mice fed fucoxanthin was lower than that in the control mice.

### LDLR, SR-B1, and ABCA1 expressions involved in cholesterol uptake and transport in the liver

**Figure 3:**
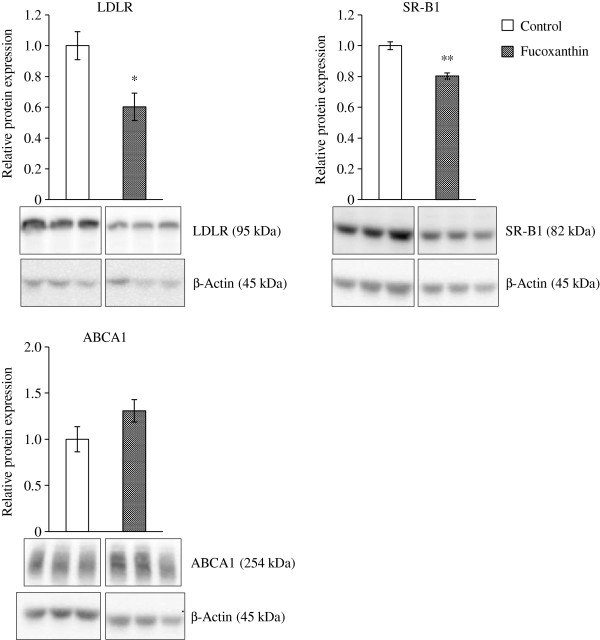
**Effect of fucoxanthin on expression levels of cholesterol transport-related proteins in the liver.** KK-*A*^*y*^ mice were fed with the AIN-93G diet with or without 0.2% fucoxanthin for 4 weeks. Liver lysates were prepared for Western blotting, and primary antibody binding was detected by appropriate secondary antibodies for chemiluminescence as described under “Materials and Methods”. Expression levels are presented relative to control group after normalization for β-Actin levels. Each value represents the mean ± standard error (n = 6). Asterisks show significant differences from the control group (* P < 0.05, ** P < 0.01).

To understand how serum cholesterol levels increased and hepatic cholesterol content decreased by fucoxanthin, we next examined cholesterol uptake-related proteins, LDLR and SR-B1, which are receptors for LDL and cholesteryl esters from HDL in the liver. LDLR expression levels in the liver were markedly decreased by fucoxanthin (Figure 3). SR-B1 expression levels were also significantly lower in the KK-*A*^*y*^ mice fed fucoxanthin than in the control mice (Figure 3). The reduced LDLR and SR-B1 expression levels in the liver indicated that fucoxanthin suppresses the uptake of LDL particles and HDL-cholesterol from the blood into the liver. On the other hand, the expression levels of ABCA1, which is a key factor for cholesterol efflux from the liver [13], were not significantly different between the control and fucoxanthin group.

### Hepatic expression of PCSK9 mRNA involved in LDLR degradation

**Figure 4:**
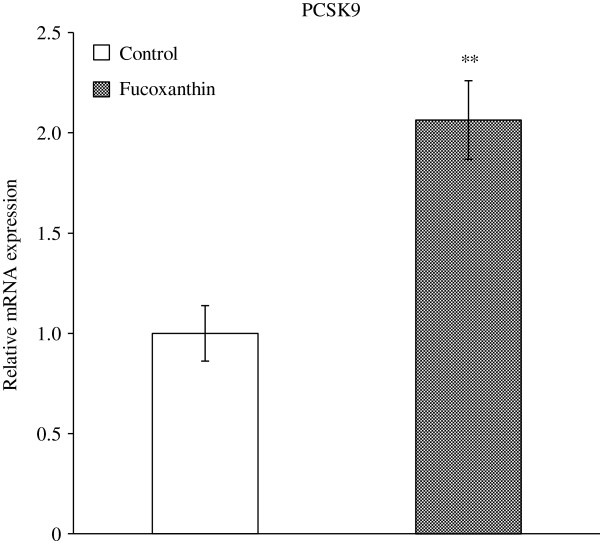
**Effect of fucoxanthin on expression levels of PCSK9 mRNA in the liver.** KK-*A*^*y*^ mice were fed with the AIN-93G diet with or without 0.2% fucoxanthin for 4 weeks. Total RNA was extracted from the liver and processed for quantitative real time PCR analysis as described under “Materials and Methods”. PCSK9 mRNA expression levels are normalized to β-actin levels. Data are presented relative to control group. Each value represents the mean ± standard error (n = 6). Asterisks show significant differences from the control group (** P < 0.01).

PCSK9, a member of the subtilisin family of serine proteases, has been shown to induce intracellular degradation of LDLR [14,15]. Fucoxanthin induced a 2-fold increase in hepatic expression of PCSK9 mRNA (Figure 4). This result suggests that reduced hepatic LDLR expression was due to PCSK9-associated LDLR degradation induced by fucoxanthin.

## Discussion

Fucoxanthin from edible brown seaweeds have reported to show anti-obesity effect on diabetic/obese KK-*A*^*y*^ mice and high-fat-fed C57BL/6N mice [7,16]. A fucoxanthin and conjugated linolenic acid-containing supplement has also been reported to induce weight loss and reduce the body and liver fat levels in obese non-diabetic women [17]. On the other hand, we and another group observed that fucoxanthin increases serum total cholesterol levels in rodents [5,6]. Cholesterol is an important component of cell membranes and is required for the biosynthesis of bile acids and vitamin D. However, an excessive serum cholesterol level, particularly LDL cholesterol, is known to be a risk factor for atherosclerosis. Therefore, we investigated the mechanisms underlying the increase in serum cholesterol levels induced by fucoxanthin.

In this study, we observed increased HDL-cholesterol and non-HDL-cholesterol levels as well as total cholesterol levels in the serum of diabetic/obese KK-*A*^*y*^ mice fed fucoxanthin. Dietary fucoxanthin also significantly increased the expression of SREBP2 and SREBP1, which are key transcriptional factors involved in up-regulation of cholesterol synthesis. Further, the mRNA expression levels of HMGCR, HMGCS, FDPS, and CYP51, which are involved in cholesterol synthesis, tended to increase in the fucoxanthin-fed mice compared with the control mice, although these differences were not statistically significant. These results show that fucoxanthin slightly enhances cholesterol synthetic pathway in the liver. In contrast, hepatic cholesterol content decreased in the fucoxanthin-diet group.

To determine how fucoxanthin regulated cholesterol metabolism we considered the balance between cholesterol biosynthesis, efflux, and its incorporation in tissues. SR-B1 is known to play an important role in selectively incorporating cholesteryl esters from circulating HDL into cells. Mice with attenuated hepatic SR-B1 expression had reduced selective HDL-cholesterol clearance and increased HDL-cholesterol levels in their blood [18,19]. Conversely, plasma HDL-cholesterol levels were dramatically decreased in mice that overexpressed hepatic SR-B1 [20]. Non-HDL-cholesterol levels, mostly LDL-cholesterol levels are determined, in part, by the rate at which LDL particles are taken up and removed from the circulation by LDLR [21]. The SR-B1 and LDLR proteins expressed in the liver at high levels and their hepatic expressions have a significant impact on circulating cholesterol levels [22]. In the present study, fucoxanthin resulted in reduced SR-B1 and LDLR expression in the liver of the KK-*A*^*y*^ mice, whereas no differences were observed in the cholesterol efflux factor ABCA1 [23]. These results show that the increased serum cholesterol levels resulted from the reduced hepatic clearance of serum cholesterol via down-regulation of SR-B1 and LDLR by fucoxanthin. In particular, the decrease in hepatic cholesterol content suggests that the reduction of incorporation of serum cholesterol may predominate over the endogenous cholesterol biosynthesis in KK-*A*^*y*^ mice.

Adipose tissue is also known to store large amounts of cholesterol and contributes to regulation of circulating cholesterol levels [24,25]. Dietary fucoxanthin suppressed the enlargement of visceral WAT during the development of obesity, which resulted in attenuation of cholesterol estimations in WAT. On the other hand, dietary fucoxanthin also increased the serum cholesterol levels in non-obese ICR mice without suppressing their body weight gain [6]. Taken together, these results suggest that the increase in serum cholesterol levels observed in the KK-*A*^*y*^ mice were partly due to the suppression of fat accumulation by dietary fucoxanthin.

Thus, we have shown for the first time that dietary fucoxanthin leads to increased HDL and non-HDL-cholesterol levels in KK-*A*^*y*^ mice through down-regulation of hepatic LDLR and SR-B1 expression, while fucoxanthin increases the expression of SREBP2, which up-regulates LDLR. As one possible mechanism, we propose that LDLR post-transcriptional regulation is involved.

Recent studies have demonstrated LDLR post-transcriptional regulation by proprotein convertase subtilisin/kexin type 9 (PCSK9) [26,27] and LXR/inducible degrader of LDLR (IDOL) signaling pathways [28]. PCSK9 is primarily expressed in the liver, small intestine, and kidneys [29]. PCSK9 binds to the EGF-A extracellular domain of LDLR and subsequently triggers its intracellular degradation in lysosomes [30]. In addition, PCSK9 overexpression in mice reduced LDLR levels and increased non-HDL-cholesterol levels [31]. Interestingly, PCSK9 has been identified as a SREBP target gene [32,33]. It is noteworthy that PCSK9 mRNA expression was up-regulated by fucoxanthin. These results suggest that fucoxanthin promotes LDLR degradation through PCSK9 mRNA up-regulation in the SREBP2 signaling pathway. Additional studies are needed to elucidate the mechanism associated with PCSK9 mRNA up-regulated by fucoxanthin.

On the other hand, it has been reported that hepatic SR-B1 expression is regulated by a variety of dietary components, hormonal, metabolic, and pharmacological manipulations [22,34]. Nuclear receptors components such as liver X receptor (LXR) and peroxisome proliferator-activated receptor-α (PPARα) are also known to regulate hepatic SR-B1 expression [35,36]. In this study, we could not identify a key factor to suppress SR-B1 by fucoxanthin. Further investigation is required to clarify the effect of fucoxanthin on SR-B1 expression.

In this study, we showed for the first time that dietary fucoxanthin resulted in increased serum HDL and non-HDL-cholesterol levels as well as total cholesterol levels via the activation of SREBP signaling and by suppressing serum cholesterol uptake in the liver via decreasing LDLR and SR-B1 expression. Further, our results suggest that LDLR degradation is promoted by fucoxanthin through up-regulation of PCSK9 and leads to increased non-HDL-cholesterol levels. These findings provide important insights on the effects of fucoxanthin on cholesterol and lipoprotein metabolism. However, it is unclear whether the responses to cholesterol metabolism are specific for rodents or common to human. Dysfunction of cholesterol metabolism is strongly associated with arteriosclerosis. Therefore, it will be necessary to further investigate the influence of high serum cholesterol levels induced by fucoxanthin on human health.

## Abbreviations

HDL: High-density lipoprotein; SREBP: Sterol regulatory element binding protein; LDLR: Low-density lipoprotein receptor; SR-B1: Scavenger receptor class B type 1; LDL: Low-density lipoprotein; PCSK9: Proprotein convertase subtilisin/kexin type 9; HMG-CoA: 3-hydroxy-3-methyl-glutaryl-CoA; CYP7A1: Cholesterol 7 α-hydroxyrase; ABCA1: ATP-binding cassette transporter A1; AIN: American Institute of Nutrition; WAT: White adipose tissue; HMGCR: HMG-CoA reductase; LSS: Lanosterol synthase; HMGCS: HMG-CoA synthase; FDPS: Farnesyl diphosphate synthase; CYP51: Lanosterol 14α-demethylase; IDOL: LXR/inducible degrader of LDLR.

## Competing interests

The authors declare that they have no competing interests.

## Authors’ contributions

FB conceived the study, its design and coordination, performed all experiments. MH participated in the design and coordination of the study and discussion of results. FB and MH discussed results and made the manuscript. YN and KM participated in the coordination of the study. All authors read and approved the final manuscript.
